# Case Report: When pancreatic cancer is not cancer: a rare case of pancreatic head paraganglioma with life-threatening delayed pseudoaneurysm bleeding

**DOI:** 10.3389/fsurg.2026.1813488

**Published:** 2026-05-07

**Authors:** Adem Ozcan, Gizem Gunes, Gulden Aydog, Gokhan Akkurt

**Affiliations:** 1 Department of Surgical Oncology, Ankara Bilkent City Hospital, Ankara, Türkiye; 2 Department of Pathology, Ankara Bilkent City Hospital, Ankara, Türkiye; 3 General Surgery Clinic, Lokman Hekim Universitesi, Ankara, Türkiye

**Keywords:** endovascular embolization, pancreatic head mass, pancreatic paraganglioma, pancreaticoduodenectomy, pseudoaneurysm

## Abstract

**Background:**

Primary pancreatic paragangliomas are extremely rare neuroendocrine tumors that may radiologically and cytologically mimic pancreatic ductal adenocarcinoma. Importantly, paragangliomas may be functional tumors capable of secreting catecholamines, which can lead to severe hypertensive crises during biopsy or surgical manipulation if unrecognized.

**Case presentation:**

A 54-year-old male presented with intermittent abdominal pain. Cross-sectional imaging revealed a 13-mm mass in the pancreatic head. ERCP-guided duodenal brush cytology suggested malignant cells, and pancreaticoduodenectomy was performed. Final histopathological examination revealed primary pancreatic paraganglioma. The postoperative course was complicated by bile leakage requiring percutaneous transhepatic biliary drainage (PTBD) and life-threatening delayed pseudoaneurysm bleeding approximately three weeks after surgery, which was successfully treated with angiographic embolization. The patient's parents were not consanguineous and were not from the same village. There was no known family history of pheochromocytoma, paraganglioma, or MEN-related endocrine disorders. However, the family history revealed several malignancies, including breast cancer in the patient's sister at the age of 55, gastric and esophageal cancer in the father during his seventies, gastric cancer in two paternal uncles and one maternal aunt, laryngeal cancer in a paternal aunt, colon cancer in a maternal uncle, and lung cancer in another maternal uncle. Given this notable family cancer history, the patient was referred to the endocrinology and medical genetics departments, and a familial cancer genetic panel was requested.

**Conclusion:**

Pancreatic paragangliomas should be considered in the differential diagnosis of pancreatic head masses. Recognizing this rare entity is important not only for diagnostic accuracy but also for perioperative safety due to the potential risk of catecholamine-related complications during invasive procedures.

## Introduction

Pancreatic ductal adenocarcinoma represents the most common malignant tumor of the pancreas. In contrast, paragangliomas are rare extra-adrenal neuroendocrine tumors arising from paraganglionic tissue and are exceptionally uncommon in the pancreas. Primary pancreatic paragangliomas account for less than 0.1% of pancreatic neoplasms, with only a limited number of cases reported in the literature (1, 2).

A major diagnostic challenge is that pancreatic paragangliomas may closely mimic pancreatic malignancies on imaging and cytologic evaluation, potentially leading to radical surgical intervention (2, 3). However, beyond the diagnostic dilemma, paragangliomas may also be functional tumors capable of secreting catecholamines, similar to pheochromocytomas. Undiagnosed functional paragangliomas may cause severe hypertensive crises during biopsy or surgical manipulation, posing a significant perioperative risk (3, 4).

Therefore, awareness of this rare entity is important not only for diagnostic considerations but also for perioperative safety and appropriate multidisciplinary evaluation.

## Case presentation

**Figure 1:**
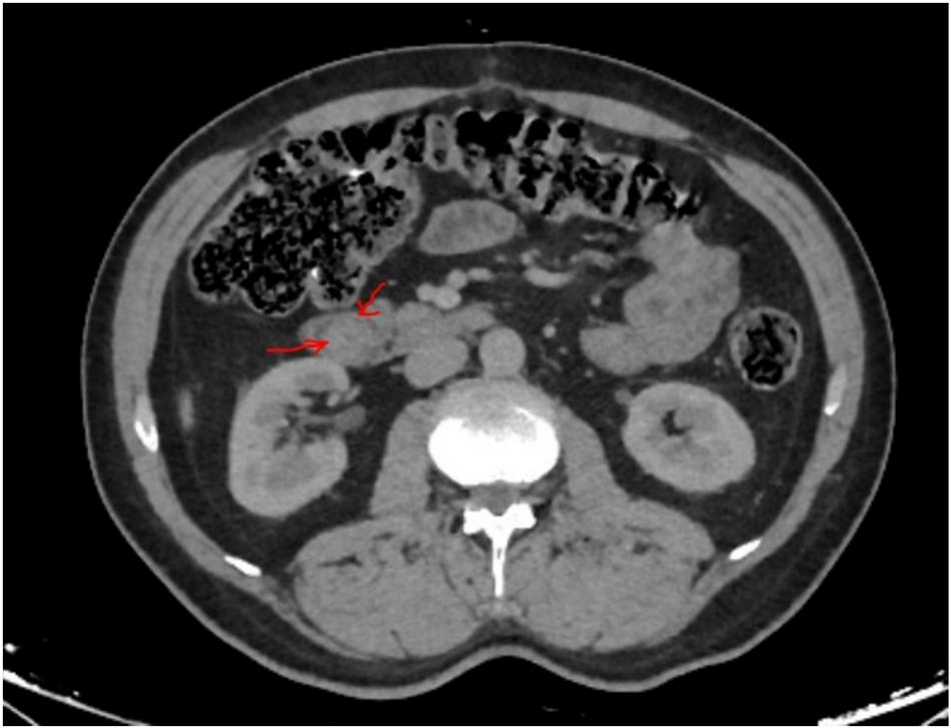
Contrast-enhanced CT demonstrating a small mass in the pancreatic head.

A 54-year-old male presented with intermittent abdominal pain without jaundice, weight loss, or systemic symptoms. Physical examination was unremarkable. Upper gastrointestinal endoscopy demonstrated minimal bulging at the ampullary region without mucosal abnormality. Contrast-enhanced computed tomography and magnetic resonance imaging revealed a hypodense 13-mm mass in the pancreatic head without evidence of vascular invasion or distant metastasis (Figure 1). Laboratory investigations, including complete blood count, biochemical parameters, inflammatory markers, and tumor markers, were within normal limits.

Endoscopic retrograde cholangiopancreatography was performed, and duodenal brush cytology demonstrated a small number of atypical malignant-appearing cells. Based on the radiologic and cytologic findings, pancreatic head malignancy was suspected, and the patient underwent pancreaticoduodenectomy (Whipple procedure).

**Figure 2:**
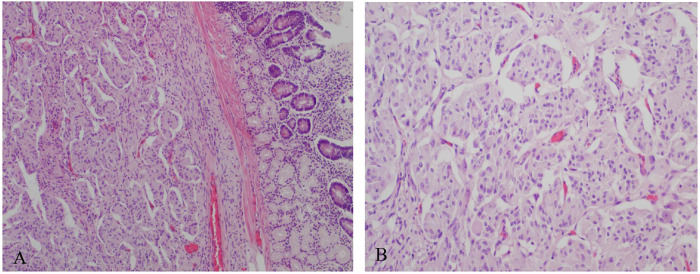
Histopathological examination showing tumor composed of epithelioid and spindle cells arranged in a nested (zellballen) pattern (H&E). **(A)** Low-power view. **(B)** High-power view.

Final histopathological examination revealed a tumor composed of epithelioid and spindle cells arranged in a nested (zellballen) pattern (Figure 2). Immunohistochemical analysis demonstrated strong synaptophysin positivity in epithelioid tumor cells and S-100 positivity in sustentacular spindle cells, consistent with the diagnosis of primary paraganglioma. Surgical margins were negative, and no lymph node metastasis was identified.

On postoperative day 6, the patient developed bile leakage from the pancreaticojejunal anastomosis, which was initially managed conservatively as a controlled fistula with external drainage. The daily drain output ranged between approximately 150–200 mL/day of bilious fluid during the initial postoperative period. The patient was closely monitored; however, due to persistent drainage and the risk of ongoing biliary leakage, percutaneous transhepatic biliary drainage (PTBD) was performed on postoperative day 10 to facilitate biliary diversion and promote fistula healing.

**Figure 3:**
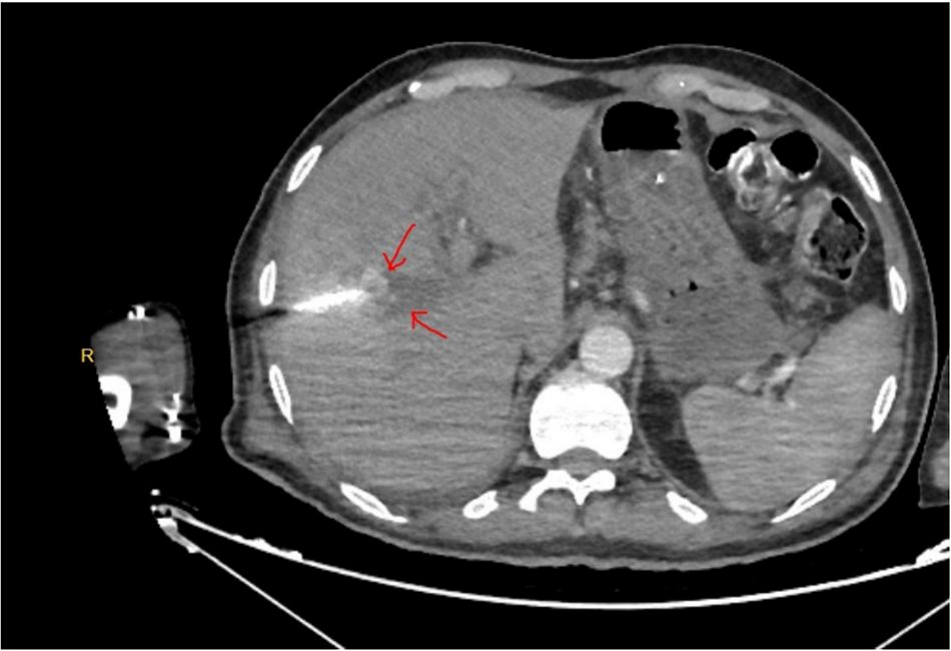
Contrast-enhanced CT angiography demonstrating active contrast extravasation from a pseudoaneurysm.

The patient was subsequently followed with the PTBD catheter for approximately 15 days. On postoperative day 25, during morning rounds, a sudden hemorrhagic output of approximately 500 mL was observed from the PTBD catheter. The patient remained hemodynamically stable. Given the clinical stability, contrast-enhanced computed tomography angiography was performed, which demonstrated active contrast extravasation from a pseudoaneurysm adjacent to the anastomotic site (Figure 3).

**Figure 4:**
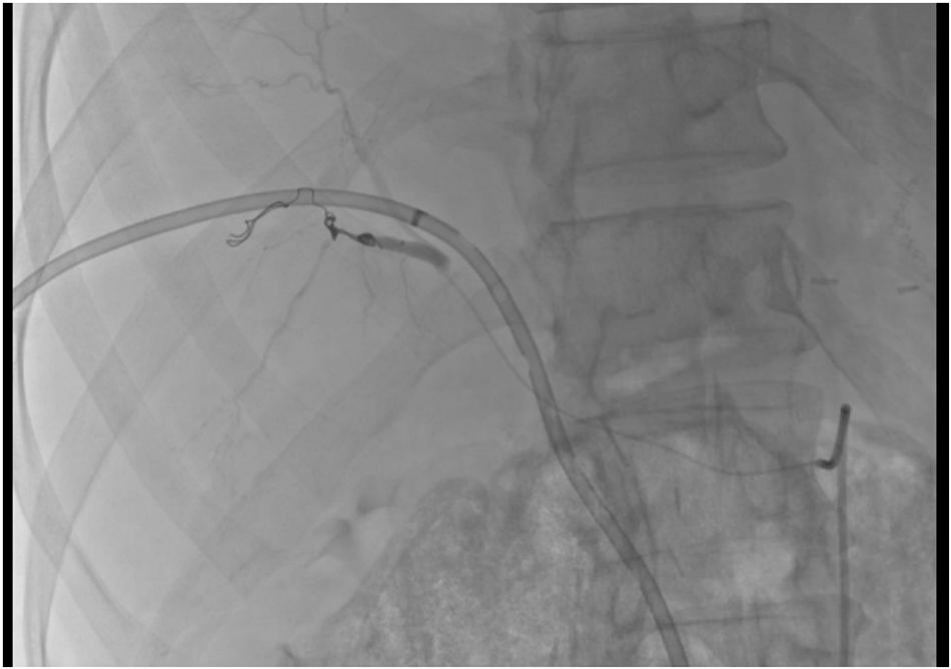
Post-embolization angiographic image demonstrating successful hemostasis after coil embolization.

Selective digital subtraction angiography confirmed the pseudoaneurysm, and endovascular coil embolization was successfully performed, achieving immediate hemostasis (Figure 4). No further bleeding episodes occurred. The biliary fistula gradually resolved under conservative management. The patient tolerated oral intake and achieved adequate glycemic control. He was discharged with outpatient follow-up after a total hospital stay of 52 days, which included postoperative management of the biliary fistula and the delayed hemorrhagic complication.

## Discussion

Primary pancreatic paragangliomas are extremely rare neuroendocrine tumors originating from extra-adrenal paraganglionic tissue. Only a limited number of cases have been reported in the literature, making preoperative recognition particularly challenging. Due to their rarity and nonspecific radiologic appearance, these tumors may easily mimic pancreatic ductal adenocarcinoma or other pancreatic neoplasms on imaging studies (1, 9).

One of the major diagnostic difficulties is the overlap between cytological findings of paragangliomas and other pancreatic tumors. Limited cytologic samples obtained via endoscopic techniques may show atypical cells that can be misinterpreted as malignant epithelial cells. Reactive atypia, crush artifacts, and degenerative cellular changes further complicate the interpretation of cytologic specimens (4, 5). Consequently, pancreatic paragangliomas may occasionally lead to a preoperative diagnosis of pancreatic malignancy and subsequent radical surgery.

Beyond diagnostic challenges, an important clinical consideration is the functional potential of paragangliomas. Similar to pheochromocytomas, some paragangliomas may secrete catecholamines. If unrecognized, invasive procedures such as biopsy or surgical manipulation may provoke severe hypertensive crises due to sudden catecholamine release (10). For this reason, awareness of paraganglioma in the differential diagnosis of pancreatic masses is important not only for diagnostic accuracy but also for perioperative safety.

Another relevant aspect of paragangliomas is their association with hereditary syndromes and germline mutations. Recent studies have shown that up to 30%–40% of paragangliomas may be associated with germline mutations, particularly involving SDHx genes (11). Therefore, patients diagnosed with paraganglioma should be considered for endocrinological evaluation and genetic counseling.

## Conclusion

Primary paraganglioma of the pancreatic head is a rare entity that may closely mimic pancreatic malignancy on imaging and cytology. Awareness of this differential diagnosis and comprehensive multidisciplinary evaluation are crucial to optimize preoperative assessment and avoid unnecessary radical surgery when feasible. Vigilant postoperative surveillance and timely endovascular intervention are essential for the management of life-threatening vascular complications such as delayed pseudoaneurysm-related hemorrhage.

This case highlights two important clinical lessons: rare neuroendocrine tumors such as pancreatic paragangliomas may closely mimic pancreatic malignancies, and unrecognized functional tumors may pose significant perioperative risks. In addition, delayed postpancreatectomy hemorrhage remains a life-threatening complication requiring rapid diagnosis and prompt endovascular management (6–8).

## Data Availability

The raw data supporting the conclusions of this article will be made available by the authors upon reasonable request, in accordance with institutional and ethical regulations, and subject to patient confidentiality requirements.
