# Supplementary material for: Case Report: When pancreatic cancer is not cancer: a rare case of pancreatic head paraganglioma with life-threatening delayed pseudoaneurysm bleeding
Source: Front Surg. 2026 May 7;13:1813488. doi: 10.3389/fsurg.2026.1813488 (PMC13190507; doi:10.3389/fsurg.2026.1813488)
Supplement: Supplementary file 1 [file Datasheet1.pdf]

CARE Checklist – Case Report

Manuscript title: When Pancreatic Cancer Is Not Cancer: A Rare Case of Pancreatic Head Paraganglioma with Life-Threatening Delayed Pseudoaneurysm Bleeding

Item	Description	Included	Location in Manuscript
Title	Includes diagnosis/intervention	Yes	Title
Keywords	2–5 keywords provided	Yes	Keywords
Abstract	Structured summary	Yes	Abstract
Introduction	Background and rationale	Yes	Introduction
Patient Information	Demographics, symptoms	Yes	Case Presentation
Clinical Findings	Physical exam, labs	Yes	Case Presentation
Timeline	Chronological presentation	Yes	Case Presentation
Diagnostic Assessment	Imaging, pathology, IHC	Yes	Case Presentation
Therapeutic Intervention	Surgery & endovascular embolization	Yes	Case Presentation
Follow-up and Outcomes	Clinical course & outcomes	Yes	Case Presentation
Discussion	Literature context, strengths/limits	Yes	Discussion
Patient Perspective	Optional	Not applicable	—
Informed Consent	Written consent obtained	Yes	Declarations

Author confirmation: We confirm that this case report adheres to the CARE guidelines and that written informed consent for publication was obtained from the patient.

Corresponding Author: Adem Özcan, MD

Institution: Department of Surgical Oncology, Ankara Bilkent City Hospital, Ankara, Türkiye
